# Primary Healthcare Quality in Conflict and Fragility: a subnational analysis of disparities using Population Health surveys

**DOI:** 10.1186/s13031-022-00466-w

**Published:** 2022-06-15

**Authors:** Marwa Ramadan, Hannah Tappis, William Brieger

**Affiliations:** 1 Present Address: Department of International Health, Johns Hopkins Bloomberg School of Public Health, Baltimore, MD USA; 2 Department of Community Medicine and Public Health, Alexandria University, Alexandria, Egypt; 3 Technical leadership and Innovations Office, Jhpiego, Baltimore, MD USA

**Keywords:** Healthcare quality, Conflict, Disparities, Proxy indicators, Primary healthcare, Central Africa, Western Africa, Sub-national

## Abstract

**Background:**

Recent global reports highlighted the importance of addressing the quality of care in all settings including fragile and conflict-affected situations (FCS), as a central strategy for the attainment of sustainable development goals and universal health coverage. Increased mortality burden in FCS reflects the inability to provide routine services of good quality. There is also paucity of research documenting the impact of conflict on the quality of care within fragile states including disparities in service delivery. This study addresses this measurement gap by examining disparities in the quality of primary healthcare services in four conflict-affected fragile states using proxy indicators.

**Methods:**

A secondary analysis of publicly available data sources was performed in four conflict-affected fragile states: Cameroon, the Democratic Republic of Congo, Mali, and Nigeria. Two main databases were utilized: the Demographic Health Survey and the Uppsala Conflict Data Program for information on components of care and conflict events, respectively. Three equity measures were computed for each country: absolute difference, concentration index, and coefficients of mixed-effects logistic regression. Each computed measure was then compared according to the intensity of organized violence events at the neighborhood level.

**Results:**

Overall, the four studied countries had poor quality of PHC services, with considerable subnational variation in the quality index. Poor quality of PHC services was not only limited to neighborhoods where medium or high intensity conflict was recorded but was also likely to be observed in neighborhoods with no or low intensity conflict. Both economic and educational disparities were observed in individual quality components in both categories of conflict intensity.

**Conclusion:**

Each of the four conflict-affected countries had an overall poor quality of PHC services with both economic and educational disparities in the individual components of the quality index, regardless of conflict intensity. Multi-sectoral efforts are needed to improve the quality of care and disparities in these settings, without a limited focus on sub-national areas where medium or high intensity conflict is recorded.

**Supplementary Information:**

The online version contains supplementary material available at 10.1186/s13031-022-00466-w.

## Background

Recent global reports [1–3] highlighted the importance of addressing healthcare quality in all settings, "leaving no one behind" as a central strategy for the attainment of sustainable development goals and universal health coverage [4]. The latter cannot be achieved without targeting the quality of care in fragile and conflict-affected situations (FCS), so that all individuals can have the highest attainable level of health, regardless of social or contextual disadvantages [3, 5].

Generally, fragile states are defined as those that are unable or unwilling to deliver essential services and protect their populations; [6] or settings where the government had lost its legitimacy and effectiveness [7]. Some studies argued on applying a specific measure or a rank to assess fragility [8], while others considered fragility a fluid concept that is difficult to measure [6]. Violent or armed conflict has been part of most fragility indices and was also viewed as a cause, manifestation, or consequence of fragility [9]. Conflict can cause a breakdown of the government's ability to carry out its functions, and vice versa: the loss of the government's legitimacy and capacity to carry out its functions can lead to more armed conflict [9].

The Institute of Medicine (IOM) defines healthcare quality as “the degree to which health services for individuals and populations increase the likelihood of desired health outcomes and are consistent with current professional knowledge” [10]. The IOM specified six main domains for the assessment of the quality of care: safety, timeliness, effectiveness, efficiency, equity, and patient-centeredness [11]. Generally, past measures had focused on effectiveness and safety more extensively than the other quality domains. Few quality measures assessed timeliness and patient-centeredness, and very few addressed equity [12].

Up to 15% of all causes of death in low- and middle-Income countries have been attributed to poor quality of care with a lost productivity cost of $1.4 to $1.6 trillion per year [2]. The situation is expected to be worse in FCS, where conflict contributes to increased mortality risk among women of reproductive age group and more than 10 million deaths of under-five children [13].

This increased mortality burden reflects the inability to provide routine services of good quality in FCS compounded by the damaged infrastructure, inadequate workforce, and other additional economic and social factors [4]. Addressing the quality of care in FCS is also challenged by the heterogeneity of contexts, with the need for context-tailored interventions [4]. Despite such heterogenicity, FCS still share the commonalities of uncertainty, volatility, limited administrative capacity, and considerable data deprivation [14].

Past reviews highlighted the limited availability of quality of care studies in humanitarian settings, and the need to focus on quality measurement strategies [5]. There is also paucity of research examining the impact of conflict on disparities in FCS [15], with mixed existing evidence. Some studies suggest that conflict can have a significant impact on inequalities both within and between countries [15, 16]; however, some argued that lower disparities can also be observed within conflict-affected countries because of a "leveling-up" or a "leveling-down" effect [16].

Conflict “levelling-up” was defined as receiving better care in areas affected by conflict due to the role of aid and international organizations pooling resources and providing services in disadvantaged settings. The latter has also been described as “islands of privilege” where displaced or refugee populations in some countries may be able to access services better than host or non-displaced populations. On the other hand, a “leveling-down” effect was described as less detectable disparities within conflict as all groups are negatively impacted by conflict [16].

In this study, we aim to address the gap in literature by examining disparities in the quality of primary healthcare (PHC) services using proxy quality of care indicators within four conflict-affected countries in sub-Saharan Africa, and to explore similarities and differences in inequities across countries.

## Materials and methods

This study uses publicly available data sources to examine disparities in the quality of PHC services in four conflict-affected countries Cameroon, the Democratic Republic of Congo, Mali, and Nigeria. These countries were selected due to the availability of globally comparable information on PHC service delivery at a time of ongoing conflict. Three equity measures were computed for each country. Each computed measure was then compared according to the intensity of organized violent events at the household cluster level the year the DHS data was collected. Additional information on country inclusion criteria and disparities metrics used are provided in an earlier study [17].

### Study contexts

The present study included four fragile states where several events of subnational organized violence and population displacement occurred over the past decade. For example, after several decades of stability, Cameroon has been dealing with insurgencies in the far North with the Boko Haram group resulting in the internal displacement of more than 500,000 people and more than 400 civilian deaths [18]. Similarly, DRC has experienced sporadic waves of violence, especially in the country's eastern part (North and South Kivu) [19], and since 2016, a new wave of violence broke out in the Kasai region in the center and the south of the country. According to UNHCR, five million internally displaced persons were recorded between October 2017 and September 2019 [20]. Mali remained fragile with insecurities and continued insurgencies by the armed groups on civilians, and UN peacekeeping operations in the northern and the central parts of the country [21]. The country also had 322,957 internally displaced persons, as of 31 January 2020 [22].

In Nigeria, ongoing insurgencies by the Boko Haram group have threatened the country’s security landscape, especially in the northern part and specifically in Borno state, which has internally displaced more than 2 million people. This is in addition to several attacks by armed groups on the oil-rich Niger delta [23].

### Data sources

The Uppsala Conflict Data Program (UCDP) and the Demographic Health Survey (DHS) databases were the primary sources of information on conflict events and the quality of care, respectively. UCDP is a globally standardized data source for organized violence [24, 25]. The primary unit of analysis in the UCDP database is an event of organized violence defined as “the incidence of the use of armed force by an organized actor against another organized actor, or against civilians, resulting in at least one direct death in either the best, low or high estimate categories at a specific location and for a specific temporal duration.” [21].

DHS are a vital source of information on population health indicators in more than 90 countries [26]. The standard DHS is conducted every five years using a large sample size (5000–30,000 households per survey) [26]. A two-stage stratified sample was utilized in the four studied contexts except for the new provinces and some parts of the established provinces in DRC, where a three-stage sample was used. Table A shows the characteristics of DHS included in the analysis (See Additional file 1).

### Quality metrics

Six consistently reported DHS indicators (along four areas of care) were utilized as proxy measures for the quality of PHC services based on the respondents’ reporting of care components. These indicators span the continuum of reproductive health, maternal health, childhood immunizations, and management of childhood illness which are critical components of a PHC system. Other PHC areas as non-communicable diseases, nutrition services, tuberculosis or mental health were not assessed due to limited availability of granular data for sub-national analysis. In the present study, we selected proxy reproductive, maternal, and child health indicators that reflect the quality of care provided and also mirror the areas of care represented in the composite coverage index (CCI). CCI has been used as a summary measure to reflect the coverage status along the four stages of the Reproductive, Maternal, Neonatal, and Child health (RMNCH) continuum of care [27, 28].

First, the quality of family planning was assessed using DHS Method Information Index (MII) or informed choice (IC) indicator. The MII has been adopted as one of the core indicators for the Family Planning 2020 initiative [29]. It is calculated based on three input questions reported by the current users of contraceptive methods: (1) whether healthcare providers informed them about other family planning methods besides the one they use; (2) whether they received information on the possible side effects of the current method; (3) and what to do if they experienced side effects [30].

Second, the quality of maternal health services was assessed using the content of antenatal care (ANC) visits. The average of six tracer items was used to measure ANC quality in the four studied countries. These tracer items were selected because they were consistently reported in the DHS surveys, and they partially fulfilled the WHO recommended elements of ANC visits [31], as well as the Primary Healthcare performance Initiative (PHCPI) tracer items for computing the provider competence in delivering ANC [32].

Next, the effectiveness and continuity of the PHC system in providing childhood immunization were assessed using patients’ reporting to construct three dropout indicators: Diphtheria-Pertussis-Tetanus (DPT)1- DPT3 dropout rate, DPT1-measles dropout rate, and Bacille Calmette–Guerin (BCG)-Measles dropout rate. The use of dropout indicators to measure the PHC system’s continuity and quality was adopted from the PHCPI framework and vital signs profile [32].

Last, management of child diarrhea with oral rehydrating therapy or continued feeding was used as a proxy measure for the quality of health education messages by providers. Caregiver adherence to providers' instruction has been proposed as a measure of effective coverage (EC) by the EC Think Tank group [33], and was also used by Nguhiu et al. to measure the quality of managing childhood illness in Kenya [34].

Additionally, we constructed a quality index as a summary measure of the overall level of PHC quality according to the following equation:$QI = \frac{{IC{ } + ANCq + \frac{{\left( {100 - {\text{``BCG-MSL}}\,{\text{d''}}} \right) + \left( {100 - {\text{``DPT1-DPT3}}\,{\text{d''}}} \right) + \left( {100 - {\text{``DPT1-MSL}}\,{\text{d''}}} \right)}}{3} + Diarrhea}}{4}$ where QI is the quality index, IC is informed choice, ANCq is receiving five components of ANC," BCG-MSL d," "DPT1-DPT3 d", and"DPT1-MSL" are BCG-measles dropout rate, and DPT1-DPT3 dropout rate, and DPT1-measles dropout rates, respectively; quality of diarrhea case management is the management of children with diarrhea according to guidelines. Except for immunization indicators, all indicators were equally weighted. Since all quality measures except immunization drop-outs were on a positive scale (the closer to 100% the better), we used non-dropouts’ rates in the construction of the quality index and the graphical presentation of variation across PHC services to avoid confusion. The detailed definition of each of the quality indicators utilized in this study is provided in supplementary methods (see Additional file 1).

### Data analysis

Household clusters were geographically and temporally linked to organized violence events. Conflict-associated fatalities located within a 50-km distance of the point representing a DHS cluster were aggregated and used to define the intensity of conflict exposure per cluster. A cut-off of more than two conflict-related deaths per 100,000 cluster population was used to differentiate medium or high-intensity conflict from no or low-intensity conflict. This cut-off point was chosen based on the World Bank definition of FCS in 2020, in which a cut-off of two deaths per 100,000 population was used to differentiate medium or high intensity conflict (beyond fragility) from other settings with low intensity conflict and/or high institutional and social fragility [35]. The denominator for estimating conflict intensity was based on the total population size per cluster according to 2015 estimates in each studied context. This has been the most recent and the closest in time to the survey conducted in the four countries. For some clusters in Nigeria (13 clusters in Borno State and one cluster in Yobe state), we could not find information on the total population size, so they were excluded from the analysis.

For each country, we first constructed the overall quality index at the national and the provincial levels; computation at the household cluster level was not possible due to the small sample size and missing data on some of the components used in constructing the index. Then, the relationship between conflict intensity and the quality of PHC services at the household cluster level was explored separately for each individual quality indicator.

Three economic and educational disparities measures were computed for each quality metric used: (1) absolute difference, (2) concentration index with Erreygers correction, and (3) coefficients of a mixed-effect logistic regression model. Additionally, we tested the interaction between conflict intensity and disparity variables using an interaction model. Detailed explanation and justification of disparities measures used are provided in an earlier study [17].

## Results

The analytic database included 85,374 women and 71,864 children living in four conflict-affected states with a total of 297,873 organized violence events and 1,702,818 associated fatalities. Table S1 provides the detailed sociodemographic characteristics of the analyzed sample (see Additional file 2).

### Sub-national variation in the quality of PHC services

**Fig. 1:**
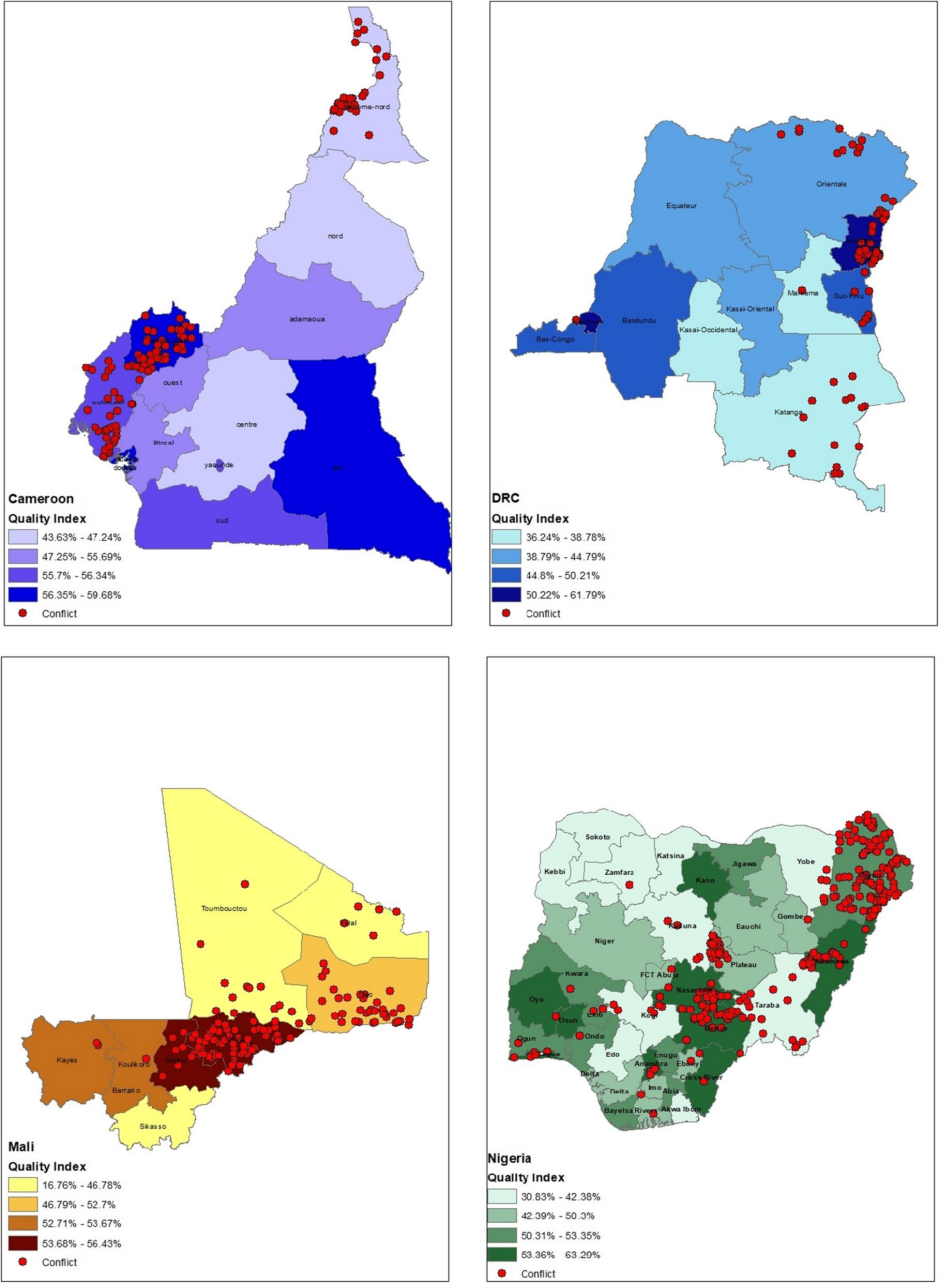
Subnational quality index in relation to conflict in Cameroon, DRC, Mali, and Nigeria

The average quality index was similar at the national level in the four studied countries ranging from 45.5% in DRC to 53.3% in Cameroon. However, sub-national variation in PHC quality was observed at the first administrative level. Mali had the most considerable sub-national variation, where the quality index ranged from 16.8% in Kidal state to 56.4% in Segou state. In DRC, the overall quality of PHC services ranged from 36.2% in Maniema province to 61.8% in the capital Kinshasa. Similarly, in Nigeria PHC quality index ranged from 30.8% in Taraba state to 63.3% in Benue state. In contrast, the lowest sub-national variation in PHC quality was observed in Cameroon, with an index ranging from 44.6% in the North region to 59.7% in the Douala region. Visually, the relationship between conflict location and the variation in the PHC quality index could not be fully established at the first administrative level (Fig. 1).

**Fig. 2:**
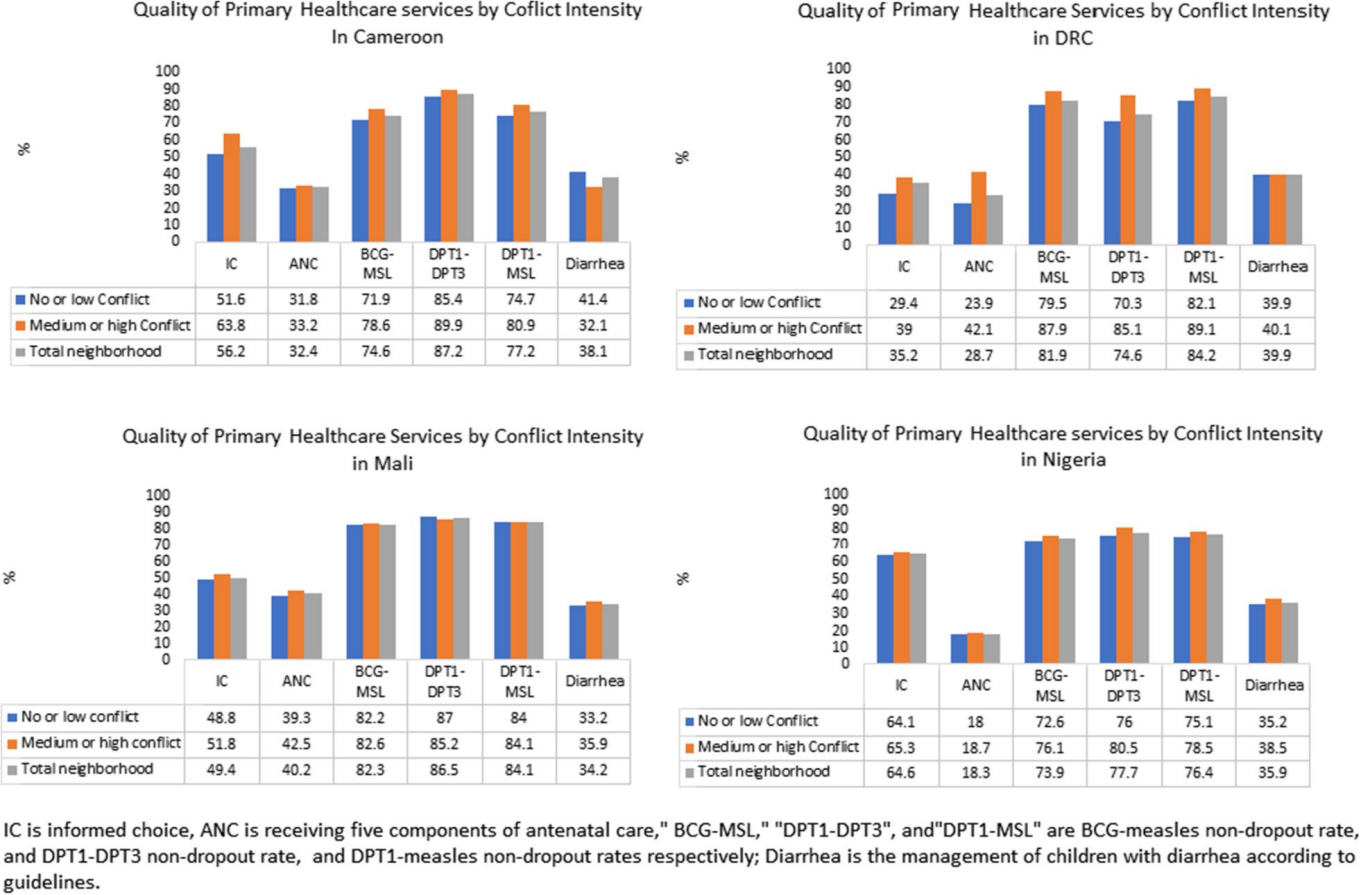
Quality of PHC services by conflict intensity in Cameroon, DRC, Mali, and Nigeria

The relationship between conflict intensity and each component of the PHC quality index was assessed at the household cluster level. Generally, both categories of conflict intensity had low quality scores in most assessed components; however, the four countries had similar or sometimes better results in the individual quality indicators in clusters surrounded by medium or high-intensity conflict compared with clusters surrounded by no or low-intensity conflict (Fig. 2). This observation was more pronounced in DRC, where five out of the six indicators constituting the PHC quality index had better results in medium or high-intensity conflict than in no or low-intensity conflict.

### Disparities in individual quality of care index components:

#### Disparities in informed choice

Overall, women with no education or in the first economic quintile were less likely to have informed choice than women with secondary or more education or in the fifth economic quintile (see Additional file 2: Tables S2 and S3). For example, Congolese women with no education had 17% less likelihood of informed choice than women with secondary or more education. Similarly, Nigerian women in the lowest economic quintile had 10% less likelihood of informed choice than women in the fifth economic quintile. Meanwhile, point estimates from the concentration index showed that disparities were generally low (concentration index < 0.10) and statistically insignificant when all sub-groups of educational or economic status are considered. Furthermore, adjusting for additional sociodemographic variables (using random intercept models) showed statistically insignificant disparities in the majority of the studied contexts. Statistically significant odds of educational disparity were only observed in Nigeria, where women with secondary or more education had 1.7 times the odds of informed choice than women with no education (*p* = 0.004). Meanwhile, statistically significant odds of economic disparity were only observed in DRC, where women in the fifth economic quintile had 12.5 times the odds of informed choice compared to women in the first economic quintile (*p* = 0.026). When considering the intensity of conflict surrounding household clusters, only Congolese and Nigerian neighborhoods had higher educational disparities in clusters surrounded by high or medium intensity conflict compared to those surrounded by no or low-intensity conflict (absolute difference = 25.1% and 17.2% vs. 9.1% and 8.1% for DRC and Nigeria respectively) (see Additional file 3: Table 1 and Additional file 4: Table 2).

Using the concentration index, no statistically significant difference in disparities was found when comparing neighborhoods by conflict intensity in any of the studied contexts (see Additional file 3: Table 1 and Additional file 4: Table 2).

Adjusting for additional sociodemographic variables using random intercept models indicated the statistical significance of educational disparities in Nigeria in both types of neighborhoods. In contrast, no statistically significant odds ratios of economic disparities were observed in any of the studied contexts. Furthermore, none of the four studied contexts had a statistically significant interaction between conflict intensity and either educational or economic disparities in informed choice (see Additional file 3: Table 1 and Additional file 4: Table 2).

#### Disparities in the quality of ANC

Generally, women with secondary or more education or in the fifth economic quintile had a better quality of ANC than women with no education or in the first economic quintile. The three measures of disparities consistently showed educational and economic inequalities in the majority of the studied contexts (see Additional file 2: Tables S2 and S3). For example, 40% of Congolese women with secondary or more education had quality ANC versus only 18% of women with no education. The concentration index also showed the aggregation of quality ANC among the most educated Congolese women (concentration index = 0.21, *p* < 0.001). Similarly, adjusting for additional sociodemographic variables showed that Congolese women with secondary education have 60% higher odds of quality ANC than women with no education (*p* < 0.001).

When household clusters were classified by conflict intensity, DRC, Mali, and Nigeria showed a higher or similar magnitude (using absolute difference) of economic and educational disparities in neighborhoods surrounded by medium or high-intensity conflict versus those surrounded by no or low-intensity conflict (see Additional file 3: Table 1 and Additional file 4: Table 2). For example, the magnitude of economic disparities among Malian women living in medium or high was 26.5% versus 19.4% in no or low-intensity conflict. On the other hand, Cameroonian women living in no or low-intensity conflict neighborhoods had a higher magnitude of economic and educational disparities in quality ANC versus those living in medium or high-intensity conflict neighborhoods (absolute difference = 11.5 vs. 25.8 and 20.0 vs. 30.6 for educational and economic disparities respectively).

When all sub-categories of educational and economic status were considered using the concentration index, the four contexts showed a statistically significant concentration of quality ANC among the most educated and the wealthiest in both types of clusters (see Additional file 3: Table 1 and Additional file 4: Table 2). Point estimates and Z tests showed a statistically significant difference between the two types of neighborhoods in Cameroon and DRC only, where lower educational and economic disparities were observed in high or medium intensity conflict neighborhoods in Cameroon (z = − 2.31, *p* = 0.021 and z = − 2.63, *p* = 0.009 respectively), while higher educational and economic disparities were observed in the medium or high-intensity conflict in DRC (z = 2.38, *p* = 0.017 and z = 3.85, *p* < 0.001 respectively) in alignment with the results obtained using the absolute difference.

In contrast, adjustment for additional sociodemographic variables in medium or high-intensity conflict (using random intercept model) showed a statistically significant odds ratio of educational disparities only in Nigeria and economic disparity only in DRC. Meanwhile, the odds ratios of educational and economic disparities were statistically significant in low-intensity conflict neighborhoods in the majority of studied contexts. Testing for interaction between conflict intensity and either educational or economic disparities was statistically insignificant in the four studied contexts (see Additional file 3: Table 1 and Additional file 4: Table 2).

#### Disparities in immunization dropouts

At the national level, children of mothers with secondary or more education or in the fifth economic quintile had lower dropout rates compared to children of mothers with no education or in the first economic quintiles, respectively, in almost all the studied contexts. For example, a considerable magnitude of economic and educational disparities in dropout rates was found in Nigeria for all dropout indicators. Dropout rates were 19.7%, 15.4%, and 17.8% for BCG-measles, DPT1-DPT3, and DPT1-measles, respectively, among Nigerian children whose mothers had secondary or more education versus 38.7%, 37.0%, and 34.4% among those with no education. Similarity dropout rates were 11.4%, 12.4%, and 10.5% among children whose mothers were in the fifth economic quintile versus 39.1%, 36.9%, and 34.8% respectively those in the first economic quintile).

When all sub-groups of educational and economic status were considered using the concentration index, a significant concentration of dropouts among the least educated or the least wealthy in Cameroon, DRC, and Nigeria was also observed. Nigeria was also the country with the largest economic and educational inequalities using the concentration index. Meanwhile, point estimates and tests of significance consistently showed the absence of either educational or economic disparities in all three types of immunization dropouts in Mali, with overall lower dropout rates compared to the other three countries.

Economic and educational disparities in dropout rates were observed in most of the studied contexts even after adjusting for additional covariates as child gender, mother age, mother employment, urban/rural status, and the number of children in the family. Nigeria was the only country consistently showing statistically significant odds ratios of both economic and educational disparities in the three dropout indicators (see Additional file 2: Tables S2 and S3).

When the intensity of conflict surrounding household clusters was considered, mothers of children living in clusters surrounded by the high-intensity conflict had similar (less than 5% difference), or higher magnitude of educational disparities in the four studied contexts and similar or higher magnitude of economic disparities in Cameroon and Mali. Meanwhile, mothers of Congolese and Nigerian children living in clusters surrounded by no or low-intensity conflict had a higher magnitude of economic disparities in dropout than those living in medium or high-intensity conflict clusters (absolute difference = 20.68 versus − 1.91 and 26.49 versus 17.74 for DRC and Nigeria, respectively (see Additional file 3: Table 1 and Additional file 4: Table 2).

When the concentration index was calculated, there was a significant aggregation of immunization dropouts only among Cameroonian and Nigerian children whose mothers were least educated or the least wealthy and living in neighborhoods surrounded by medium or high-intensity conflict. On the other hand, mothers of children living in neighborhoods surrounded by no or low-intensity conflict had statistically significant economic and education disparities, mostly in Cameroon, DRC, and Nigeria. Mali was also the only country consistently showing a lack of educational and economic disparities in the three immunization dropout indicators.

Meanwhile, no statistically significant difference was found between neighborhoods of different conflict intensity in either economic or educational disparities for immunization dropout in almost all the studied contexts (see Additional file 3: Table 1 and Additional file 4: Table 2). The only exception was observed in DRC, where economic disparities in DPT1-DPT3 dropout were statistically significantly higher in no or low intensity conflict clusters (z test = 2.48, *p* = 0.013).

Meanwhile, adjusting for additional sociodemographic variables using random intercept models revealed more statistically significant and higher odds ratios of educational and economic disparities in no or low-intensity conflict clusters than in medium or high-intensity conflict clusters. However, no statistically significant interaction between conflict intensity and educational or economic disparities existed in Cameroon, Mali, or Nigeria. Only DRC showed a statistically significant interaction between economic disparities and conflict intensity in DPT1-DPT3 dropouts (t = 2.28, *p* = 0.023), confirming the results obtained by comparing concentration indices (see Additional file 3: Table 1 and Additional file 4: Table 2).

#### Disparities in the quality of diarrhea case management

Generally, children of mothers with secondary or more education or in the fifth economic quintiles were more likely to have their diarrhea managed according to guidelines than children of mothers with no education or in the first economic quintile, respectively (see Additional file 2: Tables S2 and S3). Using absolute difference, the largest inequality gap was observed in Cameroon, where 47.4% of Cameroonian children whose mothers had secondary or more education had their disease managed according to guidelines versus 20.6% of children whose mothers had no education. Meanwhile, a narrower disparity gap was found in Mali, where children with diarrhea had an absolute difference of only 1.0% and 2.3% for educational and economic disparities, respectively. However, the overall quality score was too low to detect a meaningful difference between sub-groups.

Similarly, point estimates of the concentration index indicated higher economic and educational disparities in Cameroon and lower economic and educational disparities in Mali compared to the other studied contexts. However, adjustment for additional sociodemographic variables using random intercept models indicated the presence of statistically significant economic disparities in Mali, where children of mothers in the fifth economic quintiles had 3.0 times the odds of having their diarrhea managed according to guidelines compared to those whose mothers were in the first economic quintile (*p* = 0.041).

When the intensity of conflict surrounding household clusters was considered, children living in neighborhoods surrounded by medium or high-intensity conflict had higher levels of economic and educational disparities than children living in no or low-intensity conflict neighborhoods in Cameroon, DRC, and Mali (see Additional file 3: Table 1 and Additional file 4: Table 2). Meanwhile, Nigerian Children faced higher absolute educational disparities in low or no intensity conflict clusters versus medium or high-intensity clusters (absolute difference = 10.4 versus 0.6 respectively).

When all sub-groups of educational and economic status are considered using the concentration index, there was an aggregation of quality management of diarrhea among children whose mothers were more educated or wealthier in Cameroon and Nigeria in both types of clusters (see Additional file 3: Table 1 and Additional file 4: Table 2). Meanwhile, no or very low level of disparity was observed in DRC and Mali using a concentration index. Point estimates generally indicated higher disparities in medium or high-intensity conflict than in no or low-intensity conflict neighborhoods. However, differences were statistically insignificant except for economic disparities in Cameroon (Z = 2.3, *p* = 0.02).

Adjustment for additional sociodemographic variables also revealed similar or higher odds ratios of economic and educational disparities in medium or high-intensity conflict clusters than in no or low-intensity clusters in most studied contexts (see Additional file 3: Table 1 and Additional file 4: Table 2). A statistically significant interaction between conflict intensity and economic disparities was also observed in Cameroon (t = 3.28, P = 0.001), confirming the results obtained by the concentration index. Meanwhile, Nigeria was the only country where adjustment for additional sociodemographic variables revealed higher educational disparities in no low-intensity conflict clusters than in medium or high-intensity conflict clusters with statistically significant interaction between conflict intensity and educational disparities (t = 0.260, *p* = 0.002).

## Discussion

Despite the paucity of research documenting the impact of conflict on healthcare quality, including receiving the required components of care [36], there is a general expectation that healthcare quality will be less optimal in countries affected by conflict due to weak health systems [37–40]. The findings of the present study align with this notion. The four studied countries had an overall poor quality of PHC services, with considerable subnational variation in the quality index. Poor quality of PHC services was not only limited to neighborhoods where conflict or organized violence was recorded but was also likely to be observed in neighborhoods with no or low intensity conflict.

For example, apart from informed choice, the majority of quality indicators in Nigeria scored very low (below 40%) regardless of the level of conflict intensity. Specifically, ANC had an average quality score as low as 18% in the entire country and in both categories of conflict intensity. These findings align with previous studies in which the country had been ranked as 187 out of 200 countries in the quality of care [41]. This poor quality of care was attributed to insufficient and unevenly distributed staff, poorly equipped facilities, and lack of accountability and political commitment [42–44]. Similarly, diarrhea was managed according to guidelines in only 30–40% of sick children in the four studied countries. The latter is consistent with the findings by Clark-Deedler et al. who found that IMCI-protocols were only followed in 42% of Congolese children with severe disease [45]. Similar findings were also reported in Sub-Saharan African countries as Namibia, Kenya, Tanzania and Uganda where IMCI guidelines were followed in 40–60% in children with diarrhea [46].

In the present study, both economic and educational disparities were observed in individual quality index components regardless of conflict intensity and there was an inconsistent pattern when disparities were compared by conflict intensity across the four studied contexts. Findings of the present study as well as literature [12] suggest that three scenarios can coexist while examining disparities in conflict-affected fragile states at the subnational level. The occurrence of this scenarios depends on the disparity measure used, the indicator examined, and the studied context. In the first scenario, health inequalities are likely to be exacerbated by conflict, so more disparities can be seen in medium or high-intensity conflict than in no or low-intensity conflict. In the second scenario, medium or high-intensity conflict can be more equitable than no or low intensity conflict as a manifestation of conflict "leveling-up" or "leveling-down" effect. Interestingly, most of the situations where the second were observed were a manifestation of a conflict-leveling down effect. The latter aligns with what was described by Ranson et al. [12], where those previously advantaged become less advantaged in conflict zones resulting in a more equitable distribution of indicators in conflict. Meanwhile, the third scenario suggests that the difference in the level of disparity is unlikely to be observed when the categorization is done at the sub-national level, especially with the lack of information on population displacement.

The present study had some limitations, and caution needs to be practiced while interpreting results. First, conflict was linked to a 50-km buffer zone around the centroid of a DHS cluster, so there is a chance of misclassification due to spatial imprecision. DHS clusters are also displaced by two km in urban areas and five Km in the majority of rural areas [47], which can add more imprecision to the analysis. Using already established geographic boundaries as administrative levels is another way of classifying conflict intensity; however, we wanted to utilize the smallest available geographic unit for more granular analysis. Additionally, fragility is a fluid concept that can be difficult to measure and most literature framed fragility in relation to conflict, violence or security-related challenges as a main driver or stressor [6]. Therefore, this study only focused on sub-national conflict/organized violence as one aspect of fragility given the granularity of UCDP data and the potential impact of conflict on health systems and quality of care [37–40]. Future studies may consider analyzing disparities in relation to other fragility indices. Meanwhile, UCDP uses public sources for information on the number of fatalities, so there is a chance that conflict intensity is underestimated, meaning the cut-off used (2 deaths per 100 000 population per geographic area) would result in misclassification error. However, UCDP is the most reliable source for organized violence geolocations, and its standardized definition of armed conflict is applied across countries [48].

Analytically, we had to exclude 14 clusters in Nigeria because we could not find information on the population size, 13 of which are located in Borno state, which is the state most affected by the Boko Haram insurgency and military counterinsurgency operations that began in 2009 [49]. Therefore, there is a chance that Nigeria's conflict intensity was underestimated and, consequently, the difference between the two categories of conflict intensity may not be accurate. Additionally, cluster level weight was not approximated in DRC as we could not access census information, so the random intercept model was fitted with the assumption that all individuals within a household have an equal chance of selection, which may have affected the statistical significance of odds ratios. Meanwhile, in Cameroon, the random intercept model failed to converge in informed choice, and a flat model was fitted instead.

To the best of our knowledge, this study is the first to assess disparities in the quality of care in conflict-affected fragile states at a sub-national level. Proxy quality indicators were reconstructed across the RMNCH spectrum using population-based data. Although this approach depends on clients’ reporting of components of care rather than direct observation of the actual provided care using facility surveys, the general availability of population-based surveys in comparison to the standardized health facility surveys makes this a practical approach in understanding aspects related to the quality of care when health facility data are lacking.

Our findings suggest that quality of PHC is less optimal within the four studied FCS regardless of conflict intensity, with variations across services. Given these variations and the comprehensive nature of a PHC system, multi-disciplinary efforts are needed to improve the quality of care within FCS. The latter comes in alignment with the recent WHO action plan for addressing healthcare quality in FCS, in which they recommend a pragmatic multi-sectoral approach for the implementation and evaluation of interventions while considering the use of a practical contextualized set of indicators [4].

The results of this study also highlight the need for prioritizing quality measures within FCS as critical performance indicators. Specifically, governments, local and international organizations, and donors should consider the breakdown of these measures across different groups or social strata at the sub-national level to assess disparities.

Analytically, given the potential coexistence of multiple scenarios for health disparities within FCS, we recommend the combination of both the absolute and relative measures, while assessing disparities in healthcare quality. Our findings show that the magnitude of disparities detected by absolute measures needs to be combined with relative measures as concentration index or logistic regression to understand the distribution across the entire population as well as the pattern of disparity after accounting for other sociodemographic variables. Findings of this study also highlights the need for more mixed-methods studies to better understand the drivers of conflict, fragility and disparities within each country and the reason for variation across PHC services.

## Conclusion

The four conflict-affected countries examined in this study had an overall poor quality of PHC services with a lot of variation across the four countries. Economic and educational disparities were detected in the quality of selected PHC services, regardless of conflict intensity. Multi-sectoral efforts are needed to improve quality of care within FCS. Additionally, Stakeholders need to prioritize quality measures and their disaggregation across various social strata and economic groups. More research is needed to better understand the drivers of conflict, fragility, and disparities within countries and the reason for variation across PHC services.

## Supplementary Information

**Additional file 1.** DHS survey characteristics and detailed definitions of Individual quality indicators.

**Additional file 2: Table S1.** Sociodemographic characteristics of women and children included in the analysis by conflict intensity, **Table S2.** Educational disparities in the quality of PHC services at the national level, and **Table S3.** Economic disparities in the quality of PHC services at the national level.

**Additional file 3.** Educational Disparities in the quality of PHC services by conflict intensity.

**Additional file 4.** Economic disparities in the quality of PHC services by conflict intensity.

## Data Availability

The original datasets analyzed during the current study are available from the DHS program website, https://dhsprogram.com/data/available-datasets.cfm and UCDP Dataset Download Centre, https://ucdp.uu.se/downloads/. The analytical dataset generated during the current study is available from the corresponding author on reasonable request.

## Abbreviations

ANC: Antenatal care
BCG: Bacille Calmette–Guerin
DHS: Demographic Health Survey
DPT: Diphtheria–pertussis–tetanus
DRC: Democratic Republic of Congo
EC: Effective coverage
FCS: Fragile and conflict-affected situations
IC: Informed choice
IOM: The Institute of Medicine
MII: Method Information Index
PHC: Primary healthcare
PHCPI: Primary health care performance initiative
RMNCH: Reproductive, Maternal, Neonatal, and Child health
UCDP: Uppsala Conflict Data Program
WHO: World Health Organization
