# Supplementary material for: Primary Healthcare Quality in Conflict and Fragility: a subnational analysis of disparities using Population Health surveys
Source: Confl Health. 2022 Jun 15;16:36. doi: 10.1186/s13031-022-00466-w (PMC9202222; doi:10.1186/s13031-022-00466-w)
Supplement: Supplementary file 1 — Additional file 1. DHS survey characteristics and detailed definitions of Individual quality indicators. [file 13031_2022_466_MOESM1_ESM.docx]

**Table A: Characteristics of demographic health surveys included in the analysis.**

| **Country** | **Cameroon** | **DRC** | **Mali** | **Nigeria** |
| --- | --- | --- | --- | --- |
| **Type of Survey** | **DHS-VII** | **DHS-VI** | **DHS-VII** | **DHS-VII** |
| **Type of sample** | Two-stage  stratified sample | Three-stage  stratified sample* | Two-stage  stratified sample | Two-stage  stratified sample |
| **Total number of clusters** | 470 | 540 | 379 | 1400 |
| **Households per cluster** | 28 | 34 | 26 ** | 30 |
| **Households(n)** | 11,710 | 18,171 | 9,510 | 40,427 |
| **Interviewed**  **females(15-49)(n)** | 13,527 | 18,827 | 10,519 | 41,821 |
| **Fieldwork duration** | 06/2018-  12/2018 | 08/2013-  02/2014 | 08/2018-  11/2018 | 08/2018-  12/2018 |
| **Field restrictions** | South-West | ------- | Kidal*** | Borno |

* Two-stage stratified sample in statutory towns and cities of established provinces, three-stage stratified sample in rest of established provinces and new provinces

** 35 households per cluster was selected in Kidal, Gao, Tombouctou

*** Insecurity limited data collection in rural areas; only urban areas were visited

**Detailed definition of individual quality indicators :**

- **Informed choice:** Percentage of women aged (15-49) years currently using selected contraceptive methods who reported that they were informed about: the potential side effects or problems of the method they use, what to do in case of side effects or problems, and what other contraceptive methods they can use.
- **Quality of antenatal care:** Percentage of women aged (15-49) years with a birth in the last five years who reported receiving five components of care; having their blood pressure measured, a urine sample is taken, and a blood sample taken while receiving antenatal care for their most recent live birth in addition to being prescribed iron tablets and tablets for intestinal parasites during the pregnancy of their most recent live birth.
- **BCG-measles dropout rate:** Percent of children (12-23) months who did not receive an initial dose of measles vaccine after receiving BCG vaccine during their first year of life (according to either the vaccination card or mother's report)
- **DPT1-DPT3 dropout rate:** Percent of children (12-23) months who did not receive the three doses of DPT vaccine after receiving an initial dose of DPT vaccine (according to either the vaccination card or mother's report)
- **DPT1-measles dropout rate:** Percent children (12-23) months who did not receive an initial dose of measles vaccine after receiving an initial dose of DPT vaccine (according to either the vaccination card or mother's report)
- **Management of children with Diarrhea according to guidelines:** Proportion of children (0-59) months whose mothers had reported that their child had diarrhea in the two weeks preceding the survey, who were given the guideline-recommended treatment with oral rehydration and continued feeding.
