# Supplementary material for: Primary Healthcare Quality in Conflict and Fragility: a subnational analysis of disparities using Population Health surveys
Source: Confl Health. 2022 Jun 15;16:36. doi: 10.1186/s13031-022-00466-w (PMC9202222; doi:10.1186/s13031-022-00466-w)
Supplement: Supplementary file 2 — Additional file 2: Table S1. Sociodemographic characteristics of women and children included in the analysis by conflict intensity, Table S2. Educational disparities in the quality of PHC services at the national level, and Table S3. Economic disparities in the quality of PHC services at the national level. [file 13031_2022_466_MOESM2_ESM.docx]

| Sociodemographic characteristic | Sub-group | Cameroon | | | DRC | | | Mali | | | Nigeria | | | |
| --- | --- | --- | --- | --- | --- | --- | --- | --- | --- | --- | --- | --- | --- | --- |
|  |  | **Medium or High** | **Low or none** | **Total** | **Medium or High** | **Low or none** | **Total** | **Medium or High** | **Low or none** | **Total** | **Medium or High** | **Low or none** | **Total** |  |
|  |  | % | % | no | % | % | no | % | % | no | % | % | no |  |
| Economic quintile | Poorest | 31.88 | 68.12 | 2,001 | 8.29 | 91.71 | 4,366 | 40.63 | 59.37 | 1,868 | 21.2 | 78.8 | 7,595 |  |
|  | Poorer | 32.97 | 67.03 | 2,881 | 7.97 | 92.03 | 3,740 | 42.03 | 57.97 | 1,837 | 32.17 | 67.83 | 8,257 |  |
|  | Middle | 31.07 | 68.93 | 3,479 | 12.67 | 87.33 | 3,655 | 36.66 | 63.34 | 1,961 | 36.55 | 63.45 | 8,737 |  |
|  | Richer | 38 | 62 | 3,158 | 22.86 | 77.14 | 3,390 | 27.56 | 72.44 | 2,344 | 40.1 | 59.9 | 8,760 |  |
|  | Richest | 43.41 | 56.59 | 3,158 | 69.37 | 30.63 | 3,676 | 11.24 | 88.76 | 2,509 | 40.36 | 59.64 | 8,002 |  |
| Highest educational level | None | 33.9 | 66.1 | 2,767 | 17.55 | 82.45 | 3,357 | 34.19 | 65.81 | 6,970 | 21.04 | 78.96 | 14,062 |  |
|  | Primary | 32.11 | 67.89 | 4,301 | 14.4 | 85.6 | 7,320 | 25.02 | 74.98 | 1,371 | 40.97 | 59.03 | 6,334 |  |
|  | Secondary | 37.45 | 62.55 | 6,615 | 31.53 | 68.47 | 7,589 | 21.63 | 78.37 | 1,974 | 40.41 | 59.59 | 16,645 |  |
|  | Higher | 44.67 | 55.33 | 994 | 73.44 | 26.56 | 561 | 12.25 | 87.75 | 204 | 44.57 | 55.43 | 4,310 |  |
| Employment Status | Unemployed | 32.90 | 67.10 | 5583 | 29.26 | 70.74 | 5984 | 30.71 | 69.29 | 5493 | 28.53 | 71.47 | 14,538 |  |
|  | Employed | 37.42 | 62.58 | 9094 | 21.01 | 78.99 | 12830 | 29.67 | 70.33 | 5026 | 37.50 | 62.50 | 26,813 |  |
| Gender of children | Male | 32.95 | 67.05 | 4,938 | 19.07 | 80.93 | 9,301 | 33 | 67 | 5,052 | 31.31 | 68.69 | 17,018 |  |
|  | Female | 32.93 | 67.07 | 4,795 | 19.54 | 80.46 | 9,415 | 33.2 | 66.8 | 4,888 | 31.29 | 68.71 | 16,457 |  |
|  | Total | 32.94 | 67.06 | 9,733 | 19.31 | 80.69 | 18,716 | 33.1 | 66.9 | 9,940 | 31.3 | 68.7 | 33,475 |  |
| Age of women (years) | Mean | 30.37 | 29.53 | 29.88 | 27.54 | 28.33 | 28.09 | 29.26 | 28.17 | 28.48 | 29.34 | 28.94 | 29.08 |  |
|  | Sd | 11.86 | 11.48 | 11.65 | 9.13 | 9.31 | 9.27 | 9.10 | 9.04 | 9.07 | 9.57 | 9.64 | 9.62 |  |
| Children per woman (number) | Mean | 2.28 | 2.31 | 2.30 | 2.30 | 2.79 | 2.65 | 3.07 | 2.84 | 2.91 | 2.35 | 2.70 | 2.58 |  |
|  | Sd | 2.34 | 2.32 | 2.33 | 2.51 | 2.51 | 2.52 | 2.45 | 2.44 | 2.44 | 2.29 | 2.54 | 2.46 |  |
| Age of children  (months) | Mean | 28.73 | 29.00 | 28.90 | 28.35 | 28.98 | 28.82 | 29.16 | 28.40 | 28.63 | 29.53 | 29.44 | 29.46 |  |
|  | Sd | 17.51 | 17.24 | 17.35 | 17.35 | 17.36 | 17.36 | 17.59 | 17.20 | 17.32 | 17.42 | 17.34 | 17.51 |  |
| Total (women) | | 35.7 | 64.3 | 14,677 | 23.63 | 76.37 | 18,827 | 30.21 | 69.79 | 10,519 | 34.34 | 65.65 | 41,351 | |
| Total (children) | | 32.94 | 67.06 | 9,733 | 19.31 | 80.69 | 18,716 | 33.1 | 66.9 | 9,940 | 31.3 | 68.7 | 33,475 | |

**Table S1: Sociodemographic characteristics of women and children included in the analysis by conflict intensity in Cameroon, DRC, Mali, and Nigeria**

**Table S2: Educational disparities in the quality of PHC services at the national level**

| Indicator | Country | Absolute difference | | | Concentration Index | | Regression Coefficients | |
| --- | --- | --- | --- | --- | --- | --- | --- | --- |
|  |  | **No-education** | **Secondary+** | **P value^a^** | **CI** | **P value^b^** | **OR ^c^** | **P-value^d^** |
| Informed choice | Cameroon+ | 75.1 | 58.54 | <0.001 | 0.05 | 0.207 | 0.449 | 0.037 |
|  | DRC | 18.1 | 35.1 | <0.001 | 0.03 | 0.712 | 2.510 | 0.193 |
|  | Mali | 47.91 | 51.75 | <0.001 | 0.04 | 0.175 | 1.113 | 0.642 |
|  | Nigeria | 55.26 | 66.72 | <0.001 | 0.09 | 0.004 | 1.711 | 0.004 |
| Quality of ANC | Cameroon | 18.53 | 37.64 | <0.001 | 0.14 | <0.001 | 1.669 | 0.004 |
|  | DRC | 18.01 | 40.00 | <0.001 | 0.21 | <0.001 | 1.616 | 0.001 |
|  | Mali | 36.53 | 48.61 | <0.001 | 0.10 | <0.001 | 1.522 | <0.001 |
|  | Nigeria | 15.8 | 19.64 | <0.001 | 0.04 | <0.001 | 1.508 | <0.001 |
| BCG- measles dropout rate | Cameroon | 37.29 | 16.87 | 0.686 | -0.19 | <0.001 | 0.350 | <0.001 |
|  | DRC | 20.33 | 13.35 | 0.003 | -0.08 | <0.001 | 0.529 | 0.012 |
|  | Mali | 18.51 | 17.22 | <0.001 | -0.02 | 0.288 | 1.035 | 0.910 |
|  | Nigeria | 38.69 | 19.69 | 0.208 | -0.20 | <0.001 | 0.416 | <0.001 |
| DPT1-DPT3 dropout rate | Cameroon+ | 23.19 | 6.96 | 0.056 | -0.14 | <0.001 | 0.287 | <0.001 |
|  | DRC | 23.53 | 20.94 | <0.001 | -0.06 | 0.016 | 0.769 | 0.207 |
|  | Mali | 13.62 | 13.31 | <0.001 | 0.00 | 0.826 | 0.760 | 0.459 |
|  | Nigeria | 37.04 | 15.43 | 0.489 | -0.20 | <0.001 | 0.320 | <0.001 |
| DPT1-measles dropout rate | Cameroon | 33.13 | 15.28 | 0.915 | 0.17 | <0.001 | 0.674 | 0.373 |
|  | DRC | 16.84 | 12.11 | 0.001 | -0.06 | 0.003 | 0.509 | 0.020 |
|  | Mali | 16.75 | 15.94 | <0.001 | -0.02 | 0.319 | 1.071 | 0.835 |
|  | Nigeria | 34.4 | 17.77 | 0.103 | -0.18 | <0.001 | 0.464 | <0001 |
| Management of diarrhea | Cameroon | 20.62 | 47.43 | <0.001 | 0.22 | <0.001 | 2.382 | 0.051 |
|  | DRC | 35.43 | 39.57 | <0.001 | 0.02 | 0.622 | 1.061 | 0.754 |
|  | Mali | 34.62 | 35.62 | <0.001 | -0.01 | 0.716 | 0.994 | 0.983 |
|  | Nigeria | 32.57 | 40.44 | <0.001 | 0.08 | <0.001 | 1.324* | 0.037 |

a: adjusted Wald test of equal proportions, b: t-test assuming unequal variance, c: model was adjusted for women age, employment, urban-rural status, and number of children in the family for maternal variables and mother’s age, employment, urban-rural status, and number of children in the family and child gender for child health variables, d: t-test of random intercept model, +model failed to converge, results of fitted flat model

**Table S3: Economic disparities in the quality of PHC services at the national level**

| Indicator | Country | Absolute difference | | | Concentration Index | | Regression Coefficients | |
| --- | --- | --- | --- | --- | --- | --- | --- | --- |
|  |  | **Quintile 1** | **Quintile 5** | **P-value^a^** | **CI** | **P-value^b^** | **OR^c^** | **P-value^d^** |
| Informed choice | Cameroon+ | 55.1 | 62.31 | <0.001 | 0.09 | 0.065 | 0.774 | <0.001 |
|  | DRC | 14.8 | 38.39 | <0.001 | 0.11 | 0.104 | 12.52 | 0.026 |
|  | Mali | 42.18 | 51.74 | <0.001 | 0.04 | 0.311 | 1.538 | 0.379 |
|  | Nigeria | 60.93 | 71.97 | <0.001 | 0.11 | <0.001 | 1.444 | 0.211 |
| Quality of ANC | Cameroon | 16.5 | 42.74 | <0.001 | 0.16 | <0.001 | 3.958 | <0.001 |
|  | DRC | 16.48 | 57.51 | <0.001 | 0.28 | <0.001 | 3.805 | <0.001 |
|  | Mali | 32.27 | 47.01 | <0.001 | 0.13 | 0.000 | 1.837 | 0.009 |
|  | Nigeria | 16.19 | 18.95 | <0.001 | 0.02 | 0.022 | 1.461 | 0.001 |
| BCG- measles dropout rate | Cameroon | 37.8 | 11.65 | <0.001 | -0.19 | <0.001 | 0.422 | 0.285 |
|  | DRC | 22.24 | 13.21 | 0.033 | -0.08 | 0.002 | 0.243 | <0.001 |
|  | Mali | 17.33 | 15.58 | 0.753 | 0.00 | 0.964 | 2.047 | 0.221 |
|  | Nigeria | 39.06 | 11.44 | <0.001 | -0.22 | <0.001 | 0.196 | <0.001 |
| DPT1-DPT3 dropout rate | Cameroon+ | 22.92 | 6.42 | <0.001 | -0.11 | <0.001 | 0.446 | 0.100 |
|  | DRC | 30.59 | 14.21 | <0.001 | -0.15 | <0.001 | 0.390 | 0.012 |
|  | Mali | 13.76 | 11.76 | 0.956 | -0.01 | 0.513 | 0.139 | 0.003 |
|  | Nigeria | 36.89 | 12.42 | 0.003 | -0.19 | <0.001 | 0.282 | <0.001 |
| DPT1-measles dropout rate | Cameroon | 33.03 | 10.51 | <0.001 | -0.16 | <0.001 | 0.302 | 0.220 |
|  | DRC | 17.08 | 12.91 | 0.477 | -0.04 | 0.132 | 0.297 | 0.001 |
|  | Mali | 16.36 | 13.2 | 0.791 | -0.01 | 0.723 | 1.818 | 0.355 |
|  | Nigeria | 34.76 | 10.47 | <0.001 | -0.20 | <0.001 | 0.238 | <0.001 |
| Management of diarrhea | Cameroon | 20.35 | 51.3 | 0.011 | 0.21 | <0.001 | 3.638 | 0.093 |
|  | DRC | 32.23 | 43.25 | 0.551 | 0.07 | 0.027 | 1.217 | 0.425 |
|  | Mali | 32.33 | 34.6 | 0.060 | 0.02 | 0.637 | 3.016 | 0.041 |
|  | Nigeria | 31.78 | 52.05 | <0.001 | 0.10 | <0.001 | 1.987 | 0.004 |

a: adjusted Wald test of equal proportions, b: t-test assuming unequal variance, c: model was adjusted for women age, employment, urban-rural status, and number of children in the family for maternal variables and mother’s age, employment, urban-rural status, and number of children in the family and child gender for child health variables, d: t-test of random intercept model, +model failed to converge, results of fitted flat mode
